# Supplementary material for: Loss of full-length dystrophin expression results in major cell-autonomous abnormalities in proliferating myoblasts
Source: eLife. 2022 Sep 27;11:e75521. doi: 10.7554/eLife.75521 (PMC9514850; doi:10.7554/eLife.75521)
Supplement: Transparent reporting form [file elife-75521-transrepform1.docx]

***eLife’s* transparent reporting form**

We encourage authors to provide detailed information *within their submission* to facilitate the interpretation and replication of experiments. Authors can upload supporting documentation to indicate the use of appropriate reporting guidelines for health-related research (see [EQUATOR Network](http://www.equator-network.org/%20)), life science research (see the [BioSharing Information Resource](https://biosharing.org/)), or the [ARRIVE guidelines](http://www.plosbiology.org/article/info:doi/10.1371/journal.pbio.1000412) for reporting work involving animal research. Where applicable, authors should refer to any relevant reporting standards documents in this form.

If you have any questions, please consult our Journal Policies and/or contact us: [editorial@elifesciences.org](mailto:editorial@elifesciences.org).

**Sample-size estimation**

- You should state whether an appropriate sample size was computed when the study was being designed
- You should state the statistical method of sample size computation and any required assumptions
- If no explicit power analysis was used, you should describe how you decided what sample (replicate) size (number) to use

Please outline where this information can be found within the submission (e.g., sections or figure legends), or explain why this information doesn’t apply to your submission:

All details relating to sample size are detailed in the “Materials and Methods” section. No explicit sample size calculation was performed prior to the study design. Replicate numbers were based on previous experience of sequencing for this model.

**Replicates**

- You should report how often each experiment was performed
- You should include a definition of biological versus technical replication
- The data obtained should be provided and sufficient information should be provided to indicate the number of independent biological and/or technical replicates
- If you encountered any outliers, you should describe how these were handled
- Criteria for exclusion/inclusion of data should be clearly stated
- High-throughput sequence data should be uploaded before submission, with a private link for reviewers provided (these are available from both GEO and ArrayExpress)

Please outline where this information can be found within the submission (e.g., sections or figure legends), or explain why this information doesn’t apply to your submission:

All details relating to replicate numbers are detailed in the “Materials and Methods” section. An independent experiment is defined as an experiment carried out on a different occasion, using freshly prepared biological samples. Each sample from an individual animal was thus treated as a biological replicate. 3 biological replicates were analyzed for each condition for RNA Sequencing. For qPCR, at least 3 biological replicates were used, but technical replicates were also included (i.e. the same cDNA sample was run twice or three times on the same plate in the same set of reactions). We did not eliminate any sample data points on the outlier criterion, but reads that failed to map or mapped with quality less than 20 were removed from further analyses. All RNA-Seq data have been deposited in the ArrayExpress database at EMBL-EBI as described in the Material and Methods, *RNA Sequencing* and *Human primary adult myoblast RNA-Seq data* sections.

**Statistical reporting**

- Statistical analysis methods should be described and justified
- Raw data should be presented in figures whenever informative to do so (typically when N per group is less than 10)
- For each experiment, you should identify the statistical tests used, exact values of N, definitions of center, methods of multiple test correction, and dispersion and precision measures (e.g., mean, median, SD, SEM, confidence intervals; and, for the major substantive results, a measure of effect size (e.g., Pearson's r, Cohen's d)
- Report exact p-values wherever possible alongside the summary statistics and 95% confidence intervals. These should be reported for all key questions and not only when the p-value is less than 0.05.

Please outline where this information can be found within the submission (e.g., sections or figure legends), or explain why this information doesn’t apply to your submission:

Differential expression for genes between groups was identified based on the *DESeq2* package in R. Differential expression results are expressed based on the log2-scaled fold change, the adjusted p-values (following correction for multiple testing), and the mean FPKM across all replicates for the two groups being compared. Adjusted p-values are shown for gene ontology analyses. This is outlined in the *RNA Sequencing* section in “Materials and Methods”.

Details of other statistical tests performed throughout are outlined in the *Statistical analysis* section of the Materials and Methods. All data were first analyzed for normality using the Shapiro-Wilk test for normality, and appeared to be normally distributed. Parametric statistical tests were therefore used, where appropriate. Results for ANOVA analyses are shown with degrees of freedom (DF), F-statistic and resulting p-value for the relevant explanatory variable.

Error bars in figures represent mean ± SEM. P-values are reported as follows in figures: **p* ≤ 0.05, **p < 0.01, ****p* < 0.001, *****p* < 0.0001.

(For large datasets, or papers with a very large number of statistical tests, you may upload a single table file with tests, Ns, etc., with reference to sections in the manuscript.)

**Group allocation**

- Indicate how samples were allocated into experimental groups (in the case of clinical studies, please specify allocation to treatment method); if randomization was used, please also state if restricted randomization was applied
- Indicate if masking was used during group allocation, data collection and/or data analysis

Please outline where this information can be found within the submission (e.g., sections or figure legends), or explain why this information doesn’t apply to your submission:

Samples were allocated into experimental or control groups depending on the genotype of the animals studied. Mice used for cell isolation were chosen at random from each litter. In the Boyden chamber assay, cell count on filters was done by the experimenter, blinded to the identity of each sample. Resulting differences were further confirmed by the spectrophotometric dye measurement.

The transcription factor binding sites over-representation was assessed by comparing the lists of tested genes with 1,000 randomly selected gene promoters (selected from all genes in the mouse genome).

REVIGO was used to reduce the redundancy of GO enrichment data to rationalize the categories being compared.

This information is described in details in the “Material and Methods” sections.

**Additional data files (“source data”)**

- We encourage you to upload relevant additional data files, such as numerical data that are represented as a graph in a figure, or as a summary table
- Where provided, these should be in the most useful format, and they can be uploaded as “Source data” files linked to a main figure or table
- Include model definition files including the full list of parameters used
- Include code used for data analysis (e.g., R, MatLab)
- Avoid stating that data files are “available upon request”

Please indicate the figures or tables for which source data files have been provided:

All numerical graphs present individual datapoints to illustrate the variability.

Supplementary tables uploaded with the manuscript provide the numerical data that are represented in graphical format in the manuscript figures or as summary tables.

As described in the Material and Methods, RNA-Seq data for the mouse myoblast samples have been deposited in the ArrayExpress database at EMBL‐EBI under accession number E‐MTAB‐10322 and E-MTAB7287 for primary myoblasts and established cell lines respectively (*RNA Sequencing* section), and under E‐MTAB‐8321 for human primary myoblasts (*Human primary adult myoblast RNA-Seq data* section).
